# *Wolbachia*-Associated Bacterial Protection in the Mosquito *Aedes aegypti*

**DOI:** 10.1371/journal.pntd.0002362

**Published:** 2013-08-08

**Authors:** Yixin H. Ye, Megan Woolfit, Edwige Rancès, Scott L. O'Neill, Elizabeth A. McGraw

**Affiliations:** School of Biological Sciences, Monash University, Clayton, Victoria, Australia; Fundaçao Oswaldo Cruz, Brazil

## Abstract

**Background:**

*Wolbachia* infections confer protection for their insect hosts against a range of pathogens including bacteria, viruses, nematodes and the malaria parasite. A single mechanism that might explain this broad-based pathogen protection is immune priming, in which the presence of the symbiont upregulates the basal immune response, preparing the insect to defend against subsequent pathogen infection. A study that compared natural *Wolbachia* infections in *Drosophila melanogaster* with the mosquito vector *Aedes aegypti* artificially transinfected with the same strains has suggested that innate immune priming may only occur in recent host-*Wolbachia* associations. This same study also revealed that while immune priming may play a role in viral protection it cannot explain the entirety of the effect.

**Methodology/Findings:**

Here we assess whether the level of innate immune priming induced by different *Wolbachia* strains in *A. aegypti* is correlated with the degree of protection conferred against bacterial pathogens. We show that *Wolbachia* strains *w*Mel and *w*MelPop, currently being tested for field release for dengue biocontrol, differ in their protective abilities. The *w*MelPop strain provides stronger, more broad-based protection than *w*Mel, and this is likely explained by both the higher induction of immune gene expression and the strain-specific activation of particular genes. We also show that *Wolbachia* densities themselves decline during pathogen infection, likely as a result of the immune induction.

**Conclusions/Significance:**

This work shows a correlation between innate immune priming and bacterial protection phenotypes. The ability of the Toll pathway, melanisation and antimicrobial peptides to enhance viral protection or to provide the basis of malaria protection should be further explored in the context of this two-strain comparison. This work raises the questions of whether *Wolbachia* may improve the ability of wild mosquitoes to survive pathogen infection or alter the natural composition of gut flora, and thus have broader consequences for host fitness.

## Author Summary

*Wolbachia* is a commonly occurring bacterium or symbiont that lives inside the cells of insects. Recently, *Wolbachia* was artificially introduced into the mosquito vector dengue virus that was naturally *Wolbachia*-free. *Wolbachia* limits the growth of a range of pathogens transmitted to humans, including viruses, bacteria and parasites inside the mosquito. This “pathogen protection” forms the basis of field trials to determine if releasing *Wolbachia* into wild mosquito populations could reduce dengue virus incidence in humans. The basis of pathogen protection is not fully understood. Previous work suggests that the symbiont may activate the basal immune response, preparing the insect to defend itself against subsequent pathogen infection. Here we infect mosquitoes harbouring *Wolbachia* with a range of bacterial pathogens as a means to understand the nature of protection. We show that different *Wolbachia* strains vary in their ability to limit pathogen growth and that this correlates with the degree to which the *Wolbachia* activates the host immune response. These findings may assist with *Wolbachia* strain selection for future open field release and raise the question whether *Wolbachia* might provide a fitness advantage to mosquitoes in the wild by limiting their death due to bacterial infection.

## Introduction

*Wolbachia pipientis* is a maternally inherited intracellular bacterium that is found in a wide range of arthropod species and filarial nematodes, with approximately 40% of insect species infected [1]. *Wolbachia* spreads rapidly through populations and to high frequencies by inducing a range of manipulations of host reproduction that benefit infected females. In insects, the most common manipulation is cytoplasmic incompatibility (CI) [2], [3]. Interestingly, some *Wolbachia* strains that cannot induce reproductive manipulations still spread through populations [4]. This would not be predicted unless there were other positive benefits for *Wolbachia*-infected insects. Despite numerous laboratory and semi-field based experiments examining a range of life history traits, few studies have identified significant fitness benefits of infection [5], [6], [7], [8]. Most reveal no effect [9] or weak negative effects [10], [11], [12]. It is possible, however, that there are benefits to *Wolbachia* infections that are only detectable under field conditions or in circumstances not yet tested in the laboratory.

Recently, *Wolbachia* was found to either extend the lifespan and/or increase the survival of Drosophila infected with native viruses, a trait termed pathogen protection [13], [14], [15]. Subsequently, *Wolbachia* strains native to Drosophila have also been shown to confer pathogen protection against arboviruses, bacteria, filarial nematodes and the malaria parasite *Plasmodium gallinaceum* when stably transinfected into the mosquito *Aedes aegypti* [16], [17] [13], [17], [18], [19], [20], [21], [22]. This broad-based pathogen protection may offer a potential fitness advantage, assisting cytoplasmic incompatibility in the maintenance and spread of *Wolbachia* in wild populations. Understanding the true fitness effects of *Wolbachia* infections in mosquitoes is important as these symbiont-infected mosquitoes are being released into wild populations as part of a biocontrol strategy for reducing dengue virus transmission to humans [23].

While the mechanism of pathogen protection is not fully understood, several recent studies have shed some light on its basis. It was originally hypothesized that priming of the insect immune response might provide a single mechanistic explanation for symbiont-induced protection against viruses, bacteria, nematodes and malaria. Under an immune priming model, *Wolbachia* infections activate the basal immune response, better preparing insects against subsequent infection by pathogens. Three different *A. aegypti:Wolbachia* strain associations have been created thus far and in each case infection with the symbiont induces the host immune response [18], [19], [20]. The same is true for transient infections established in the mosquito *Anopheles gambiae* [22]. Immunity genes upregulated in these mosquitoes include members of the opsonisation, Toll and melanisation pathways [18], [21], [24]. Whether the expression of this limited set of insect immunity genes can confer protection against pathogens other than bacteria [25] is not clear, although the Toll pathway participates in dengue virus control [26] and the Imd, Toll, opsonisation and melanisation pathways assist in Plasmodium control [18], [27], [28], [29], [30].

In each case where *Wolbachia*-associated immunity gene activation has been reported, the host insects did not have histories of association with this symbiont. In Drosophila naturally infected with *Wolbachia* there is no activation of the immune response and no bacterial protection [31], [32]. There is, however, weak protection against dengue virus [24] as well as other native viruses [13], [14] indicating that innate immune priming cannot explain viral protection in this host. Interestingly, in *A. aegypti* transinfected with the same *Wolbachia* strains native to *D. melanogaster*, there is both innate immune priming and strong protection against dengue virus. The comparative study indicates that innate immune priming alone cannot fully explain pathogen blocking although it may be contributing to the strength of the effect in *A. aegypti* [24], [33]. This same study also revealed that *Wolbachia* strains differ in their level of immune induction in *A. aegypti* [24]. The *w*MelPop *Wolbachia* strain, known for causing life shortening and other fitness effects in its host, is present in more tissues and grows to higher densities [16], [20], [34], [35] and is associated with a greater immune response than the *w*Mel strain, which is present in fewer tissues and grows to much more moderate densities [17].

While the transcriptional profiles of *Wolbachia*-infected *A. aegypti* predict that they should experience broad protection against bacterial infection, evidence of bacterial protection in this host comes from a single study demonstrating the ability of *w*MelPop to protect against systemic *Erwinia carotorovra* infection [21]. Here we expose *A. aegypti* stably transinfected with either the *w*Mel or the *w*MelPop strain to infection with several bacterial pathogens using in previous infection studies in *D. melanogaster*. We characterised the response to two extracellular bacteria, *E. carotovora* [36] and the slow-killing but highly pathogenic *Burkholderia cepacia* [37], and two intracellular bacteria, *Salmonella typhimurium* [37], [38] and the slow-growing *Mycobacterium marinum* [37]. Following pathogen infection we then examined mosquito survival and corresponding changes in *Wolbachia* and pathogen densities. As a control, we also confirm that these *Wolbachia* strains provide no protection against these same pathogens in *D. melanogaster*. We studied that the protective effect of *w*MelPop and *w*Mel in terms of both survival and delayed death rates. We examined the association between survivorship and pathogen load. Our result indicates either a direct effect of immune priming on the symbiont or an energetic tradeoff, with sick hosts affecting resources for *Wolbachia's* growth and replication.

## Materials and Methods

### Ethics statement

Approval for blood feeding by human volunteers for maintenance of the mosquito colony was granted by the Monash University Human Research Ethics Committee (2007001379). Volunteers provided written informed consent to participate.

### Fly and mosquito

The *w^1118^* fly line infected with *w*Mel (*w* ^1118^ *w*Mel) or *w*MelPop (*w* ^1118^ *w*MelPop) and their respective tetracycline-cured lines (*w* ^1118^ *w*Mel.tet and *w* ^1118^ *w*MelPop.tet respectively) were used in this study [34], [39]. PCR using primers specific for the *w*Mel and *w*MelPop IS5 repeat was used to confirm the tetracycline-cured lines to be free of *Wolbachia* [40]. Flies were reared on standard yellow corn meal medium at 25°C with 50% relative humidity and a 12:12 hr light/dark cycle. Around fifty individuals were allowed to oviposit in bottles with 40 ml of fly food for two days. After eclosion, adults were transferred to and aged in vials at a density of ∼30 individuals per vial.

Mosquito lines used in this study are laboratory lines artificially infected with *w*Mel (MGYP2) or *w*MelPop-CLA (PGYP1) and their tetracycline-cured (PGYP1.tet and MGYP2.tet respectively) *Wolbachia* uninfected counterparts [16], [17]. Mosquitoes were reared under standard laboratory conditions (26±2°C, 12:12 hr light/dark cycle, 75% relative humidity). Mosquito larvae were fed 0.1 mg/larvae of TetraMin Tropical Tablets once a day at a density of 150 larvae per 3 liters of distilled water in trays. Adults were transferred to cages (measuring 30×30×30 cm) at emergence at 400 individuals per cage. Adults were supplied with a basic diet of 10% sucrose solution.

### Bacterial culture

*E. carotovora* strain 15 (*Ecc15*) and *S. typhimurium* strain TM11 were cultured in LB medium in a shaker at 37°C [36], [37]. *B. cepacia* clinical isolate AH1345 was cultured in brain heart infusion broth (Oxoid, Australia) at 37°C in a shaker [37], [38]. *M. marinum* was cultured at 29°C in the dark without shaking in Middlebrook 7H9 broth (Difco, Australia) supplemented with OADC [41].

### Survival assay

For survival assay, female flies and mosquitoes aged 3–8 days were used. Insects were anesthetized with CO_2_ before being infected by either stabbing with a needle previously dipped into a bacterial culture or injected with 69 nl via an individually calibrated pulled glass needle attached to a Nanoject II injector (Drummond Scientific Company, Broomall). PBS mock stabbed or injected insects were used as control for the infection processes.

For *E. carotovora* and *M. marinum* infection, bacterial cultures were pelleted (OD∼20). Flies were infected by injection in the abdomen and mosquitoes were infected by pricking the thorax [36], [42]. For *B. cepacia* infection, flies and mosquitoes were infected by pricking in the thorax from a bacterial culture of OD = 0.1 measured spectrometrically at 600 nm [37]. For *S. typhimurium* infection, bacterial culture of OD of 0.1 at 600 nm was injected into flies and infection in mosquitoes was achieved by pricking in the thorax [37], [38]. Survival data were collected over the entirety of the insect's life, however, only the first 200 hours post infection were used for analysis (when over 90% of death had occurred) prior to the onset of shortening effects of *w*MelPop. Survival curves were analyzed using Kaplan-Meier analysis, and log-rank statistics (SPSS statistics version 19, SPSS Inc, an IBM Company) were corrected for false positives using q-value [43].

### Bacterial density quantification

We used qPCR to quantify bacterial density as it is a more sensitive and specific way to estimate bacterial number than plating for bacterial growth, especially for bacteria that are difficult to culture [44]. Specific primers (Table 1) were designed for the bacterial 16S ribosomal RNA gene of each of the bacterial pathogens using Primer3 [45]. For *Wolbachia* previously published primers for the single copy ankyrin gene WD0550 were employed [46]. Bacterial gene copy numbers were expressed as a ratio by normalizing against copy numbers for the host rpS17 [47] gene (Table 1). To correct for potential differences in body size between different mosquito lines that would affect host rpS17 copy number, the change in bacterial density was expressed as the fold increase of 16S/rpS17 ratio post-infection to the 16S/rpS17 ratio immediately after infection (zero hour post-infection). Post-infection mosquitoes were collected at either 8 or 26 hours when ∼10% of the individuals had died. Five pairs of females were used for each bacterial strain.

**Table 1:** Primers used in qPCR determination of bacterial density.

Gene	Genbank ID	Forward primer (5′-3′)	Reverse primer (3′-5′)	Product size (bp)
*A. aegypti* rpS17	AAEL004175-RA	CACTCCCAGGTCCGTGGTAT	GGACACTTCCGGCACGTAGT	81
*E. carotovora* 16S	AB681950.1	CAGCCACACTGGAACTGAGA	GTTAGCCGGTGCTTCTTCTG	204
*B. cepacia* 16S	AB681702.1	ACGCCCTAAACGATGTCAAC	AGGATTCCGACCATGTCAAG	202
*S. typhimurium* 16S	EF489442.1	TGGAAACGGTGGCTAATACC	CTCAGACCAGCTAGGGATCG	143
*M. marinum* 16s	AB636134.1	TTCATGTCCTGTGGTGGAAA	GTGCAATATTCCCCACTGCT	181
*W. pipientis* WD0550	AY649751.1	CAGGAGTTGCTGTGGGTATATTAG	TGCAGGTAATGCAGTAGCGTAAA	74

DNA was extracted from individual mosquitoes using DNeasy spin columns (QIAGEN, Australia) and qPCR was performed on LightCycler 480 (Roche Applied Science, Australia) using PlatinumSYBRGreen (Invitrogen Inc, Carlsbad, CA) according to manufacturer's instructions. For each reaction a mastermix of 2 µl RNase-free water, 5 µl of SYBR Supermix and 0.5 µl of each primer (5 µM) was added to 2 µl of DNA. The cycling protocol was as follows: 1 cycle Taq activation at 95°C for 2 minutes, 40 cycles of denaturation at 95°C for 5 s, annealing at 60°C for 5 s, extension at 72°C for 15 s, fluorescence acquisition 78°C, and 1 cycle of melt curve analysis from 68–95°C in 1°C steps. A standard curve was constructed using serially diluted DNA to calculate the amplification efficiency of each set of primers. The raw output data of crossing points (CP) was normalized by taking into consideration the differences in amplification efficiency of target and the reference genes using Q-Gene [48]. Scatter plot with median ± interquartile range were plotted. Treatment effects were then examined using Mann-Whitney *U* tests using Statistica 8.0 (StatSoft, Inc.).

## Results

### *Wolbachia* does not protect against bacterial infection in flies

**Figure 1:**
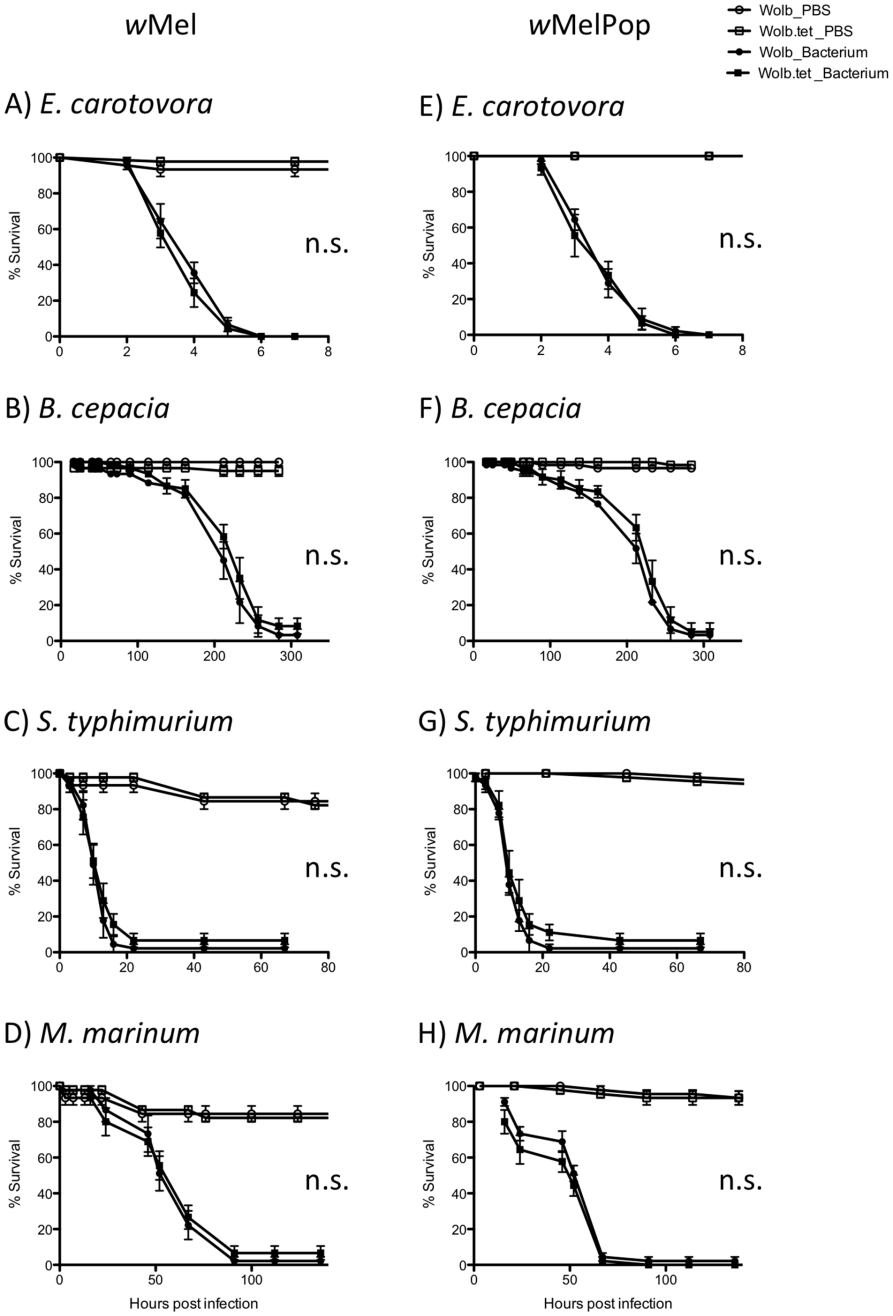
Survival curves of *Wolbachia*-infected (circle) and *Wolbachia*-uninfected (square) Drosophila infected with pathogenic bacteria (solid) or mock infected with PBS (open). (A&E) *E. carotovora*, (B&F) *B. cepacia*, (C&G) *S. typhimurium* and (D&H) *M. marinum*. Error bars are SEM calculated from the three replicates. * P-value<0.05, ** P-value<0.01, *** P-value<0.001 denote differences in survival between *Wolbachia* infected and uninfected lines by Log-rank statistics (Table S1A).

We tested whether *w^1118^w*Mel and *w^1118^w*MelPop fly lines were protected against either extracellular or intracellular bacterial infection by comparing their survival to that of their tetracycline-cured counterparts. The pathogens varied in their virulence as measured by how quickly they killed flies. Almost all the flies infected with *E. carotovora* and *S. typhimurium* were dead within 24 hours, whereas those infected with *B. cepacia* and *M. marinum* survived for several days. There was no significant difference in survival, however, between *w^1118^w*Mel and *w^1118^w*Mel.tet or between *w^1118^w*MelPop and *w^1118^w*MelPop.tet for any of the pathogens tested (Figure 1A–H, Table S1A).

### *Wolbachia* provides variable protection against bacterial infection in mosquitoes

**Figure 2:**
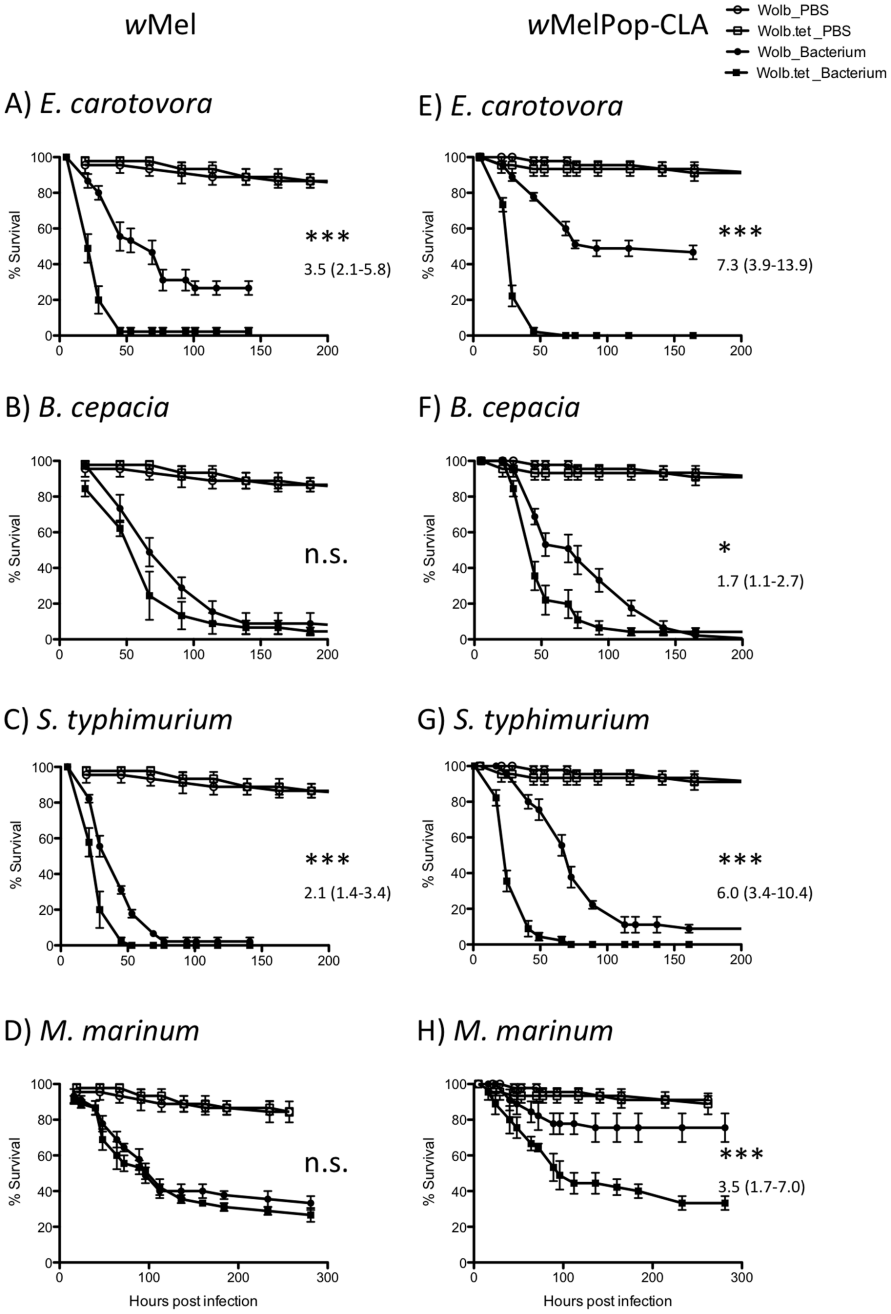
Survival curves of *Wolbachia*-infected (circle) and *Wolbachia*-uninfected (square) mosquitoes infected with pathogenic bacteria (solid) or mock infected with PBS (open). (A&E) *E. carotovora*, (B&F) *B. cepacia*, (C&G) *S. typhimurium* and (D&H) *M. marinum*. Error bars are SEM calculated from the three replicates. * P-value<0.05, ** P-value<0.01, *** P-value<0.001 denote differences in survival between *Wolbachia* infected and uninfected lines by Log-rank statistics (Table S1B). The relative risk ratio [EXP(B)] of *Wolbachia* uninfected to infected lines with 95% confidence intervals shown in parentheses is reported on graphs where significant.

We examined mosquitoes infected with *w*Mel (MGYP2) or *w*MelPop-CLA (PGYP1) for protection against the four bacterial strains. After demonstrating no significant difference in survival between *Wolbachia*-infected and uninfected mosquitoes when injected with PBS (Table S1B), direct comparisons were then made between *Wolbachia*-infected vs uninfected mosquitoes in the presence of each of the pathogens. Infection with wMelPop-CLA provided protection against all four pathogens, but *w*Mel protected only against *E. carotovora* and *S. typhimurium* (Fig. 2, Table S1B). For these two pathogens, the relative risk ratios (risk of dying for *Wolbachia*-uninfected relative to *Wolbachia*-infected individuals in the presence of the pathogen) were also greater for PGYP1 compared to MYGYP2 (Fig. 2, A vs E, C vs G) although only significantly so for *S. typhimurium* (Table S1B).

The nature of the protection when present also varied. In response to *E. carotovora* (Fig. 2A & E), *Wolbachia* conferred both a delay in death (lines not parallel) and an increase in survival from 0% to 28% and 0% to 50% for *w*Mel and *w*MelPop-CLA, respectively. The *w*Mel strain only delayed death for *S. typhimurium*-infected mosquitoes (Fig. 2C) and the same was true for *w*MelPop-CLA mosquitoes infected with *B. cepacia* (Fig. 2F). The *w*MelPop-CLA strain delayed death and increased survival from 0 to 10% for *S. typhimurium* infected mosquitoes at 200 hours post-infection and from 38% to 79% for those infected with *M. marinum* at 287 hours post-infection (Fig. 2 G&H). Taken together these patterns demonstrate that, compared to *w*Mel, *w*MelPop-CLA offers mosquitoes protection against a broader range of pathogens, greater strength of protection, and is more likely to provide increased survival rather than simply delaying death.

### *Wolbachia* infection leads to reductions in pathogen densities

**Figure 3:**
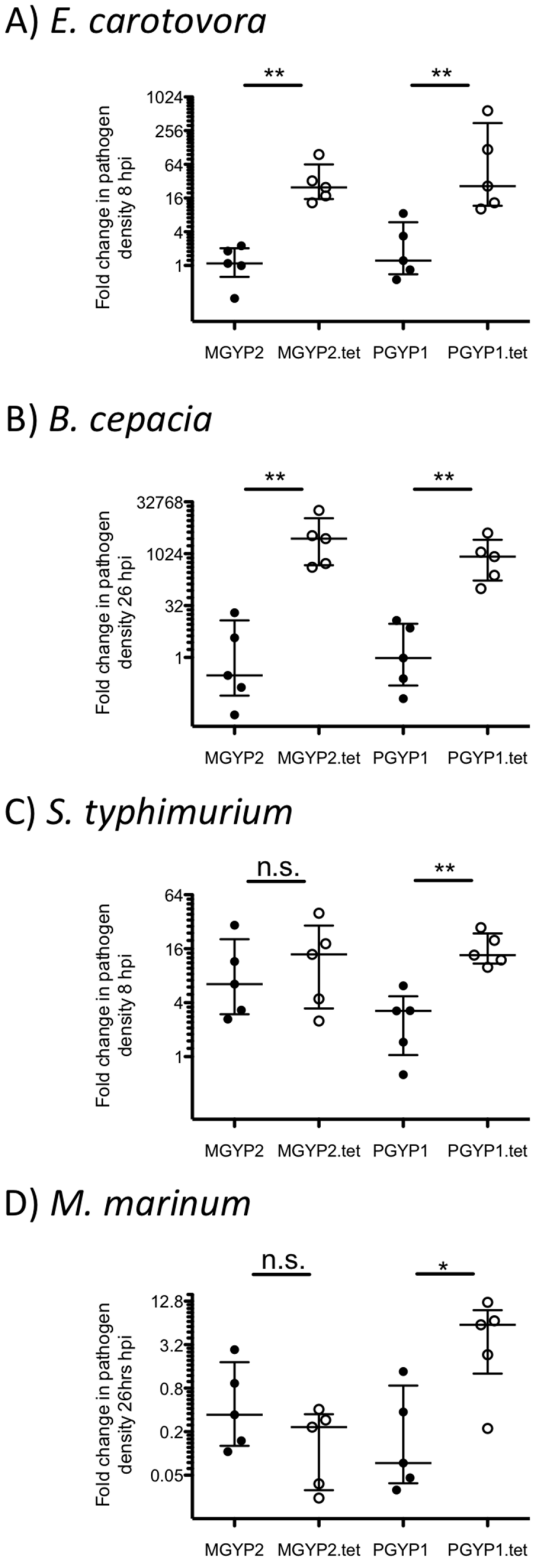
Median (with interquartile range) fold change in pathogen density variable hours post infection (hpi) in mosquitoes. Five pairs of individuals were used for *E. carotovora* (A), *B. cepacia* (B), *S. typhimurium* (C) and *M. marinum* (D). (Mann-Whitney U-test; * P-value<0.05, ** P-value<0.01).

To investigate if co infection with *Wolbachia* could limit pathogen replication, we used qPCR to quantify the change in the bacterial density during early infection. For the extracellular bacteria *E. carotovora* (Figure 3A) and *B. cepacia* (Figure 3B), both *w*Mel and *w*MelPop-CLA infected mosquitoes were able to inhibit the bacteria, with pathogen densities significantly higher in *Wolbachia*-uninfected counterparts relative to infected. This difference in pathogen density appears to be correlated with increased survival and reduced death rate due to *E. carotovora* (Fig. 2 A&E) but less so for *B. cepacia* (Fig. 2B & F). In contrast, only *w*MelPop-CLA infection results in reduced densities of the two intracellular pathogens (Fig. 3 C&D). For both *S. typhimurium* and *M. marinum*, as for *E. carotovora*, reduction in the proliferation of intracellular bacteria correlates with significant delays in mosquito death and increases in survival (Fig. 2 C–D, G–H). The magnitude of the reduction in pathogen density due in association with *Wolbachia* was also more modest for intracellular bacteria (∼3–4 fold) than for extracellular infections (∼30–7000 fold).

### Pathogen infection decreases *Wolbachia* density in mosquitoes

**Figure 4:**
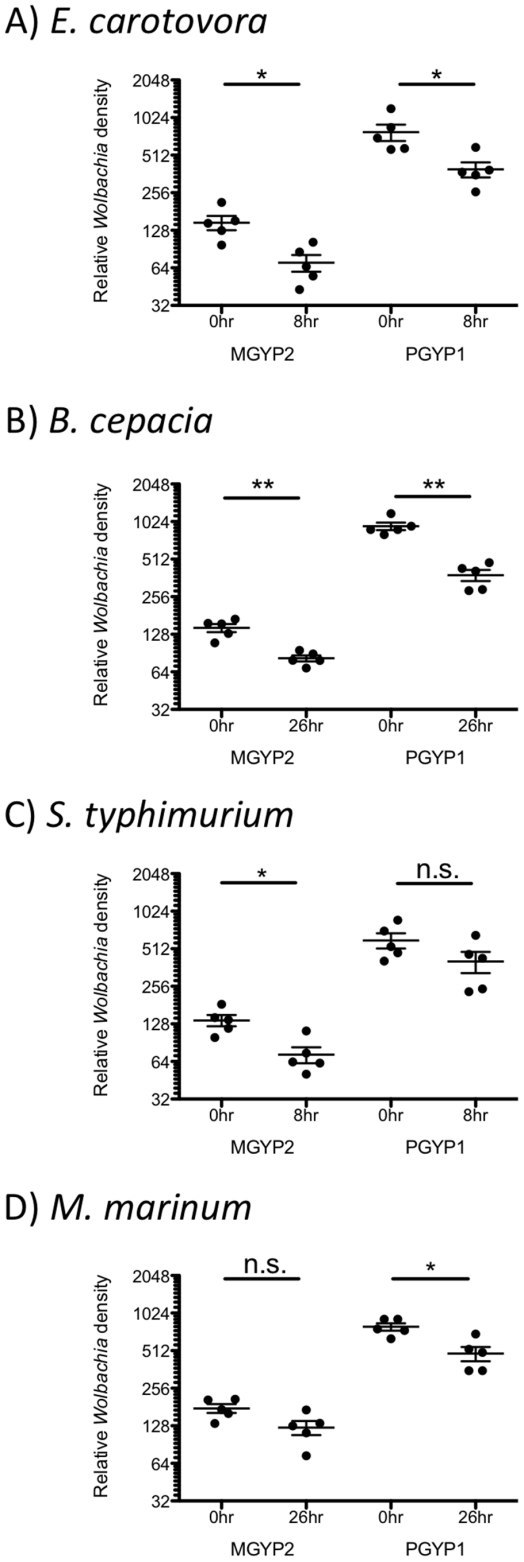
Median (with interquartile range) relative *Wolbachia* density after infection in mosquitoes. Five pairs of individuals were used for *E. carotovora* (A), *B. cepacia* (B), *S. typhimurium* (C) and *M. marinum* (D). (Mann-Whitney U-test; * P-value<0.05, ** P-value<0.01).

To investigate whether the presence of pathogenic bacteria could affect the replication and/or survival of *Wolbachia*, we used qPCR to quantify the change in *Wolbachia* density during the first 8 hours of infection. In most cases, *Wolbachia* densities were significantly reduced during the early hours of infection with a pathogen regardless of *Wolbachia* strain (Fig. 4). Fold reductions were similar across all pathogen x *Wolbachia* strain pairings, ranging from 1.5–2.7. Only *w*MelPop-CLA in response to *S. typhimurium* and *w*Mel in response to *M. marinum* did not experience statistically significant reductions (Fig. 4 C & D), although the median *Wolbachia* densities demonstrate decreasing trends.

## Discussion

In agreement with previous reports [31], [32], we found no evidence that natural *Wolbachia* infection confers bacterial protection in Drosophila. This result is expected under a model where symbiont priming of the innate immune response leads to the production of antimicrobial peptides. Our assays were carried out in 3–8 day old Drosophila, which is also the age range for which previous transcriptional profiling showed no evidence of immune activation [24]. If protection correlates with *Wolbachia* densities, however, it is possible that protection could occur in older age Drosophila harboring *w*MelPop. This strain over-replicates, causing increasing host cell lysis with age and hence greater contact with effectors of the host immune system [34]. Given the life-shortening phenotype caused by the virulent *w*MelPop, however, it becomes increasingly challenging to test for protection in older insects and to disentangle death due to symbiont effects from death due to the pathogen. It is becoming increasingly evident that immune induction in mosquitoes is results from the recent introduction of *Wolbachia*. Protection against virus in Drosophila, which is independent of immune activation, may be explained by other mechanisms such resources competition and the natural microbiota of the host [24], [33].

We also hypothesized that the *w*MelPop-CLA strain would provide greater protection in mosquitoes than *w*Mel, given the strain's propensity to colonise a wider array of somatic tissues and to replicate to higher densities [17], [20]. The nature of *w*MelPop-CLA-induced protection was indeed broader, operating against all four bacterial pathogens, compared to the two against which *w*Mel offered protection. The strength of *w*MelPop-CLA's protection was also greater, conferring higher survival and longer delays in time to death than *w*Mel. These patterns are in line with predictions from the innate immune gene expression profiles of *A. aegypti* infected with the two strains. Both *w*Mel and *w*MelPop-CLA induce expression of a set of genes representing the following classes: antimicrobial peptides (initiated by both Toll and IMD), melanisation, Toll pathway constituents, C-type lectins, serine proteases and Transferrin [24]. In almost all cases, the level of induction was much greater in response to *w*MelPop. *E. carotovora*, *B. cepacia* and *S. typhimurium* are known to be sensitive to the action of the IMD pathway and the AMPs it produces [49] and so differences in the transcriptional control of these pathways in response to *w*Mel versus *w*MelPop-CLA could be responsible for the variation in protection. There were also aspects of the transcriptional response that were unique to *w*MelPop-infected mosquitoes. Up-regulation of NF-kappaB Relish-like transcription factor and IMD pathway signalling gene, AAEL007624 (3.4 fold) and the AMP, *cecropin* AAEL015515 (69 fold) occurred only in *w*MelPop-CLA infected mosquitoes [24], and this could also have played a role in conferring better protection against these pathogens.

Perhaps the most noticeable group of immune genes showing strong induction in the presence of *w*MelPop-CLA but not *w*Mel are prophenoloxidase genes (AAEL011763, AAEL010919, AAEL014837, AAEL006877 and AAEL011764), exhibiting upregulation of greater than 30 fold [24]. These prophenoloxidase genes are known to be involved in melanisation, opsonisation and encapsulation of bacteria [50] and may have contributed to the differential protection against all pathogens. Melanisation may be the only aspect of innate immune priming effective against *M. marinum*, as mutations in the Toll or IMD pathway in Drosophila do not affect its survivorship when infected with *M. marinum* [41].

In most cases, protection was associated with reduction of pathogen densities. This indicates that in general *Wolbachia* provides true resistance to infection in mosquitoes, rather than simply tolerance, where bacteria continue to replicate but their pathogenic effects on hosts are mitigated [51]. In the case of *B. cepacia*, both *Wolbachia* infections reduced pathogen replication, but *w*Mel did not provide protection and *w*MelPop-CLA delayed death only slightly. This pathogen is highly virulent [52], able to avoid the melanisation response [37] and, like its close relative *Pseudomonas aeruginosa*, may require only a few bacteria to kill insects [53], [54].

The *w*MelPop-CLA strain was better at limiting densities of intracellular pathogens than *w*Mel. It is not clear if this is because wMelPop-CLA is triggering greater AMP production [24] that would operate on intracellular pathogens when they are in the extracellular environment, or if it differentially induces aspects of immunity specific to the intracellular environment. Recognition receptors that operate in the extracellular environment are well-characterised for insects, including Peptidoglycan binding proteins (PGRPs) and Gram negative binding proteins (GNBP) but few if any equivalents for the intracellular environment have been described [55]. The only candidate receptor for the intracellular space is PGRP-LE given its lack of secretion signal and Toll-independent activation of autophagy leading to the control of *Listeria monocytogenes* infections [56], [57]. Expression of the PGRP-LE homolog (AAEL013112) in *A. aegypti*, however, was not upregulated by either *w*Mel or *w*MelPop-CLA in mosquitoes [24].

For *M. marinum* while there is control of pathogen densities, the magnitude of infection densities remains small relative to the other pathogens. This may be due to how *M. marinum* colonises insects, first establishing itself inside hemocytes with little sign of bacterial growth before spreading systematically and causing tissue damage [41].

Lastly, in nearly all cases, *Wolbachia* densities declined during pathogen infection. This may be the direct result of innate immune effectors elicited by the pathogens in addition to those elicited by *Wolbachia*. Fold reductions in *Wolbachia* numbers are in keeping with those of the intracellular pathogens that would be exposed to the same aspects of the immune response. A related study in the mosquito *A. albopictus* naturally infected with *Wolbachia* also reported reductions in symbiont density after co-infection with the vectored virus Chikungunya [58]. Alternatively, *Wolbachia* reductions may spring from indirect effects of innate immune priming. Mounting an immune response with the production of AMPs and prophenoloxidases is costly [59]. Infection by intracellular pathogens also carries with it the added cost of direct competition for resources within cells. *Wolbachia* is highly dependent on its host for nutrition and replication [60] and as such co-infection with pathogens may cause *Wolbachia* replication to slow due to resource limitation. Because change in *Wolbachia* numbers was measured over short time periods (8–26 hrs) and because estimates of *Wolbachia's* dividing time are long (∼14 hours) [61], however, our data are more likely to provide support for control of densities by the direct effect of the immune response on *Wolbachia*.

While this study uses the transcription of the inducible immune response in adult insects to interpret patterns of *Wolbachia*-associated bacterial protection, the approach may not capture other relevant aspects of immunity. At least one study has shown the ability of *Wolbachia* infection to affect hemocyte count [62]. This constitutive aspect of immunity, defined early in development will continue to have real effects on the performance of phagocytosis in the adult [63]. Also, the recent transinfection of *Wolbachia* into new insect hosts has been associated with increases in autophagy [64] and the generation of reactive oxygen species [65], both of which may not be captured in transcriptional measures.

Our findings have the following implications for use of *Wolbachia* as a biocontrol agent in *A. aegypti*. Firstly, different *Wolbachia* strains may vary substantially in the immune priming they induce. As the efficacy of *Wolbachia* is being trialled as a dengue control agent around the world, one of the main decision points going forward will be which strain(s) to deploy. A full understanding of the strain-based differences in pathogen protection and fitness effects will aid in that decision. Secondly, bacterial protection may be affecting mosquito fitness in the field. Recent studies have shown that gut flora can play a role in insect nutrition [66], behaviour [67] and ability to vector pathogens [26]. It is possible that innate immune priming may be altering the composition of the gut microbiome. Priming may also provide protection against natural infections in the wild and assist with spread and maintenance of the symbiont. A field-based assessment of the performance of *w*Mel and *w*MelPop with respect to native pathogen control is in order although it is difficult to sample rare and sickly insects in wild populations with systemic bacterial infections [68].

Lastly, immune priming induced by *Wolbachia* may also provide a mechanistic explanation for protection against *Plasmodium gallinaceum*, as there appears to be greater evidence of Imd, Toll, opsonisation and melanisation involvement in control of this parasite than there is for viruses [18], [27], [28], [29]. Dengue represents the test case for use of *Wolbachia* for pathogen protection. Given that malaria cases outnumber dengue by at least 10-fold [69], the potential rewards for developing the symbiont for malaria vectors are great [70]. As the technical challenges around infecting the host are solved [71], the need to understand the basis of pathogen blocking becomes immediate [22], [72].

### Conclusions

Our findings support previous studies indicating that native *Wolbachia* infections in *D. melanogaster* do not confer pathogen protection against bacteria. In the recently transinfected *A. aegypti*, in contrast, we demonstrate pathogen protection that varies by strain, with *w*MelPop-CLA exhibiting more effective protection than *w*Mel against a broader range of bacteria. We also provide evidence that the expression of innate immunity genes induced by *Wolbachia* infection in mosquitoes likely explains these differences in protection. Future work will need to identify the potential role for innate immune priming as an enhancer of viral protection, assess whether bacterial protection is providing benefit for mosquitoes in the field. These findings may assist with *Wolbachia* strain selection for field release.

## Supporting Information

Table S1 Adjusted P-Values of log-rank statistics (Mantel-Cox) comparing the effect of *Wolbachia* infection or the *Wolbachia* strain on survival of bacterial infection in A) flies and B) mosquitoes. * P-value<0.05, ** P-value<0.01, *** P-value<0.001.
(DOCX)
