# Supplementary material for: Wolbachia-Associated Bacterial Protection in the Mosquito Aedes aegypti
Source: PLoS Negl Trop Dis. 2013 Aug 8;7(8):e2362. doi: 10.1371/journal.pntd.0002362 (PMC3738474; doi:10.1371/journal.pntd.0002362)
Supplement: Table S1 — Adjusted P-Values of log-rank statistics (Mantel-Cox) comparing the effect of Wolbachia infection or the Wolbachia strain on survival of bacterial infection in A) flies and B) mosquitoes. * P-value<0.05, ** P-value<0.01, *** P-value<0.001. (DOCX) [file pntd.0002362.s001.docx]

| A |  |  |  |  |
| --- | --- | --- | --- | --- |
|  | *Wolbachia* infected vs. uninfected | | *w^1118^w*Mel vs. *w^1118^w*MelPop | |
|  | *w^1118^w*Mel | *w^1118^w*MelPop | *Wolbachia* uninfected | *Wolbachia* infected |
| PBS control stabbed | 0.5914 | 0.8345 | 0.5914 | 0.8345 |
| PBS control injected | 0.9703 | 0.9900 | 0.2992 | 0.4058 |
| *E. carotovora* | 0.5914 | 0.8425 | 0.8425 | 0.9900 |
| *B. cepacia* | 0.2992 | 0.3878 | 0.9703 | 0.9900 |
| *S. typhimurium* | 0.5914 | 0.4058 | 0.9900 | 0.8425 |
| *M. marinum* | 0.8425 | 0.5914 | **0.0474*** | 0.5072 |
|  |  |  |  |  |
|  |  |  |  |  |
| B |  |  |  |  |
|  | *Wolbachia* infected vs. uninfected | | MGYP2 vs. PGYP1 | |
|  | MGYP2 | PGYP1 | *Wolbachia* uninfected | *Wolbachia* infected |
| PBS control stabbed | 0.9391 | 0.9900 | 0.8345 | 0.5914 |
| *E. carotovora* | **0.0000***** | **0.0000***** | 0.2952 | 0.1355 |
| *B. cepacia* | 0.2304 | **0.0122*** | 0.2992 | 0.9900 |
| *S. typhimurium* | **0.0001***** | **0.0000***** | 0.9703 | **0.0000***** |
| *M. marinum* | 0.8345 | **0.0008***** | 0.8425 | **0.0018**** |
|  |  |  |  |  |
